# Effect of caffeine intake on self-reported and genetic prediction of osteoarthritis: an epidemiological study and Mendelian randomization analysis

**DOI:** 10.3389/fnut.2024.1405161

**Published:** 2024-07-17

**Authors:** Zhongkai Ji, Yucheng Shen, Dong Chen, Zhidong Liu, Jiuming Dai, Bin Dai, Wei Deng

**Affiliations:** Department of Orthopedic Surgery, Binhai County People's Hospital, Yancheng, China

**Keywords:** caffeine intake, osteoarthritis, population-based study, Mendelian randomization analysis, NHANES

## Abstract

**Background:**

Osteoarthritis (OA) holds the distinction of being the most widespread musculoskeletal disorder. Any disruptions in the integrity of the articular cartilage can result in joint malfunction, discomfort, and impaired physical functioning. Increasing evidence indicates the negative impacts of caffeine intake on hyaline cartilage. The primary objective of this study was to delve deeper into understanding the potential link between the consumption of caffeine and the risk of developing OA.

**Methods:**

In this study, we constructed logistic regression models to evaluate the correlation between caffeine consumption and the risk of osteoarthritis using data from the National Health and Nutrition Examination Survey. Following that, we utilized genome-wide association studies to conduct a Mendelian randomization (MR) analysis investigating the association between coffee consumption and the likelihood of developing knee OA. We employed various statistical methods, including inverse variance weighting (IVW), weighted median, weighted mode, simple mode, and MR-Egger regression, to ensure comprehensive analysis and robust conclusions. To evaluate heterogeneity and the potential impact of pleiotropy, we conducted several statistical tests, including Cochran's Q test, MR-Egger intercept test, MR Pleiotropy RESidual Sum and Outlier test (MR-PRESSO), and MR Steiger test.

**Results:**

The weighted multivariate logistic regression analysis showed that the relationship between high caffeine intake (95–206 and ≥206 mg/day) and OA prevalence remained significantly high even after adjusting for covariates using the lowest caffeine intake (< 11 mg/day) as reference: Model 1—OR (95% Cl) = 1.365 (1.18–1.58) and 1.59 (1.38–1.83); Model 2—OR (95% Cl) = 1.21 (1.04–1.42) and 1.44 (1.23–1.68); and Model 3—OR (95% Cl) = 1.19 (1.01–1.40) and 1.30 (1.10–1.52), respectively (*p* < 0.05). The findings from the fixed effects inverse variance weighted (IVW) analysis revealed a statistically significant link between coffee intake and the likelihood of developing knee osteoarthritis: OR = 1.94; 95% confidence interval (Cl) =1.471–2.517; (*p* < 0.001). Consistent findings were obtained across various other methods, including MR-Egger regression, weighted median, weighted mode, and simple mode analyses.

**Conclusion:**

Our study showed a positive correlation between OA prevalence and high caffeine intake (≥95 mg/day).

## 1 Introduction

Osteoarthritis (OA) is the most common chronic rheumatic disease worldwide and is the leading cause of disability in middle-aged and elderly people (1–8). It is characterized by progressive articular cartilage degeneration that eventually leads to joint damage (8). An estimated 250 million people have OA, and OA-induced symptoms such as pain, stiffness, and loss of function can lead to increased personal dependence, which is associated with economic costs (1, 8, 9). Currently, most OA research is focused on knee OA (10, 11). Moreover, symptomatic radiographically confirmed OA of the knee is more prevalent than that of the hip, affecting approximately 16% and 10%, respectively of a sample population aged ≥45 years (12).

Caffeine is a naturally occurring methyl flavonoid found in coffee, cocoa beans, tea, and cola nuts. Consequently, it is present in various beverages and food products, such as coffee, tea, soft drinks, energy drinks, cocoa, and chocolate. The Kantar Worldpanel Beverage Consumption Survey assessed the caffeine intake of 37,602 caffeinated beverage consumers (aged ≥2 years, representing the entire US population) and found that 85% of Americans consume at least one caffeinated beverage per day, with coffee being the main source of caffeine intake for all age groups (13). Studies have reported that coffee consumption is associated with an increased risk of chronic diseases, such as depression, type 2 diabetes, Parkinson's disease, rheumatoid arthritis, and OA (14–18). In addition to these effects, multiple *in vitro* and *in vivo* experiments indicate that excessive intake of caffeine may be detrimental to the musculoskeletal system, including articular cartilage. The potential impact of caffeine on articular cartilage has been clearly demonstrated in rodent animal models. In these experiments, prenatal caffeine exposure (PCE) at doses lower than clinical toxic levels significantly affected the integrity of fetal articular cartilage, falling within the range of exposure experienced by some pregnant women. Specifically, there is ample evidence suggesting that caffeine plays a role in the pathophysiology of both articular and growth plate cartilage, and is associated with abnormal bone growth resulting from alterations in growth plate cartilage. Thus, caffeine intake is implicated in severe changes to articular cartilage, which is related to the development of osteoarthritis (19–27). Some studies also suggest a link between caffeine intake and knee osteoarthritis, but specifically in males rather than females (14). However, the samples used in these previous studies lacked national representativeness, or were conducted too early to be indicative of current circumstances. Additionally, there has been no research assessing the causal relationship between caffeine intake and osteoarthritis from the perspective of genetic variations.

Mendelian randomization (MR) analysis is becoming increasingly important in assessing potential causal relationships between different exposures and clinical outcomes. While observational associations may be confounded by extraneous factors or reverse causation, genetic associations in Mendelian randomization are distinct; they are not influenced by these issues because genetic variation is randomly distributed at conception (28). This random allocation aids researchers in more reliably disentangling the effects of specific exposures on outcomes. Furthermore, the random allocation and independent assortment of genetic variation enable Mendelian randomization (MR) analysis to mitigate the impact of confounding variables (29). This is achieved by utilizing genetic markers as instrumental variables (IVs) for the investigated exposures. In doing so, MR analysis effectively separates the effects of these exposures from potential confounders, thereby enhancing the reliability of causal inference (30). In this study, we conducted a cross-sectional investigation using the extensive National Health and Nutrition Examination Survey (NHANES) database to explore the potential association between caffeine intake and the risk of developing osteoarthritis (OA). Through logistic regression, we confirmed the relationship between caffeine intake and osteoarthritis. Subsequently, we employed Mendelian randomization (MR) analysis using large-scale genome-wide association study (GWAS) data to further validate the causal relationship between caffeine intake and the occurrence of osteoarthritis, focusing particularly on genetic-level evidence.

## 2 Materials and methods

### 2.1 Cross-sectional study

#### 2.1.1 Study design

NHANES, supported by the National Center for Health Statistics, is a comprehensive and regular program designed to gather health-related data from a nationally representative sample of non-institutionalized individuals in the USA. NHANES encompasses a multifaceted approach, comprising interviews that capture essential details regarding demographics, socioeconomic factors, dietary patterns, and health-related indicators. Additionally, it incorporates comprehensive physical examinations, which encompass medical, dental, and physiological assessments, all conducted by trained healthcare professionals. Furthermore, laboratory tests are administered to further augment the data collection process.

#### 2.1.2 Study participants

In this study, we used the NHANES data from 2009 to 2016 and only included respondents (aged ≥ 20 years) with self-reported OA (*N* = 19,402). All the participants with incomplete data on caffeine intake (*n* = 522), marital status (*n* = 5), education level (*n* = 13), poverty–income ratio (PIR) (*n* = 1,353), drinks (*n* = 1,988), body mass index (BMI) (*n* = 900), diabetes (*n* = 8), hypertension (*n* = 22), and Patient Health Questionnaire (PHQ)-9 score (*n* = 11) were excluded from the study. After exclusions, a total of 14,580 participants were included in this analysis (Supplementary Figure S1).

#### 2.1.3 Exposure variable

In this study, the exposure variable was “caffeine intake.” Detailed dietary intake information, including the types and quantities of food and beverages (including all types of water) consumed in the 24-h period preceding the interview, was gathered through in-person interviews conducted in private rooms as part of NHANES. Afterward, the energy and nutrient content, including caffeine, of each product were determined using the Food and Nutrient Database for Dietary Studies provided by the United States Department of Agriculture. This allowed for the estimation of patients' daily caffeine intake in milligrams (mg). This data was used to represent the daily caffeine intake of patients in this study, after excluding the patients on a special diet. Caffeine intake was classified into four quartiles, namely Q1: < 25^th^ percentile, Q2: 25^th^-50^th^ percentile, Q3: 50^th^-75^th^ percentile, and Q4: ≥75^th^ percentile, with Q1 as the reference category. Total coffee consumption (mg/day) was classified into four groups, namely Group 1: < 11 mg/day, Group 2: 11–95 mg/day, Group 3: 95–206 mg/day, and Group 4 ≥206 mg/day. The detailed data can be accessed in the Total Nutrient Intakes, First Day of Dietary Interview (https://wwwn.cdc.gov/nchs/nhanes/Default.aspx).

#### 2.1.4 Outcome variable

In this study, the outcome variable was “OA.” The OA patients were identified based on their answers to the following two questions: “Has a doctor ever told you that you had arthritis?” (answer: “yes”) and “Which type of arthritis was it?” (answer “osteoarthritis”). Self-reported information is considered reliable for common chronic conditions, as demonstrated by an 85% agreement between self-reported osteoarthritis and clinically well-defined osteoarthritis (31).

#### 2.1.5 Covariates

A total of 13 confounding factors were included in this study. Age was considered as a continuous variable. Gender (male/female), race (Mexican American/other Hispanic/non-Hispanic black/non-Hispanic white/other race–including multi-racial), education level (under high school/high school or equivalent/college or AA degree/College Graduate or above), marital status (married/living with partner/widowed/divorced/separated/never married), PIR (< 1.3, 1.3–3.5, or >3.5), smoking status (smoked or did not smoke at least 100 cigarettes in lifetime), drinking status (had or did not have at least 12 alcoholic drinks per year), physical activity (resulting in a significant increase in breathing or heart rate for at least 10 consecutive minutes during the week; categorized as “yes” or “no”), PHQ-9 score (cut-off value: ≥10 indicating depression), BMI (normal: < 25 kg/m^2^, overweight: 25–30 kg/m^2^, or obese: ≥30 kg/m^2^), and history of hypertension and diabetes (self-reported physician diagnosis) were considered as categorical variables.

#### 2.1.6 Statistical analysis

Normally distributed continuous variables were reported as mean ± standard deviation and compared by Student's *t*-test and non-normally distributed continuous variables were reported as median (interquartile range) and compared by Mann–Whitney U test. Categorical variables were represented as absolute values (percentages) and compared by χ^2^ test. All statistical analyses were performed using R statistical software v4.3.1, and a two-sided *p-*value < 0.05 was deemed statistically significant. The relationship between caffeine intake and OA prevalence was explored using three consecutive multivariate logistic regression models. Model 1 was non-adjusted; Model 2 was adjusted by adding age, gender, race, and education level; and Model 3 was adjusted for Model 2 by adding BMI, physical activity, drinking status, smoking status, diabetes, hypertension, and PHQ-9 score. In statistical analysis, restricted cubic splines are a type of spline function used to model the relationship between a predictor variable and an outcome variable in a flexible and non-linear manner. They are often employed in regression analysis when the relationship between the predictor and outcome variables is not linear. By using restricted cubic splines, researchers can capture complex relationships and non-linear patterns in the data more effectively than traditional linear models. The “restricted” aspect refers to constraints placed on the spline function to ensure smoothness and stability, typically by limiting the number of knots or control points used in the spline. The dose-response association was examined using restricted cubic spline (RCS) analysis with three knots located at the 10th, 50th, and 90th percentiles. Non-linearity was examined by analysis of variance. Hence, This study employs Restricted Cubic Spline (RCS) methodology using segmented regression to calculate the linear threshold inflection point, modeling the continuous variable of coffee consumption. Additionally, logistic regression is employed to analyze the categorical variable of caffeine intake.

### 2.2 MR study

#### 2.2.1 Sources of two-sample MR

UK Biobank is a comprehensive biomedical database and research resource that houses extensive genetic and health information from 500,000 British participants. The genome-wide association study (GWAS) summary dataset for coffee intake (ukb-b-5237) sourced from UK Biobank comprises more than 428,860 samples of individuals with European ancestry. In addition, we obtained aggregated data on knee osteoarthritis (ebi-a-GCST007090) from a prior study, which included 24,955 participants of European ancestry.

#### 2.2.2 Selection of IVs for coffee consumption

In order to develop genetic tools for assessing coffee consumption and its association with osteoarthritis (OA), we identified single nucleotide polymorphisms (SNPs) that demonstrated high reliability (*P* < 5 × 10^−8^) and independence from caffeine intake (r^2^ < 0.001, with a distance of 10,000 kb). We abstained from utilizing SNP proxies and set the minimum allele frequency at 0.01. The strength of individual SNPs was assessed by calculating the F-statistic, with a threshold of F-statistic >10, which is currently recognized as indicative of the SNP's capacity to sufficiently mitigate potential bias. Furthermore, SNPs associated with the outcome by conducting separate searches in PhenoScanner to identify potential confounders associated with osteoarthritis were excluded. Additionally, SNPs that were subsequently associated with the results were further eliminated using MR Pleiotropy RESidual Sum and Outlier (MR-PRESSO). Finally, the SNPs associated with coffee consumption were identified and retained as IVs.

#### 2.2.3 Statistical analysis

**Figure 1:**
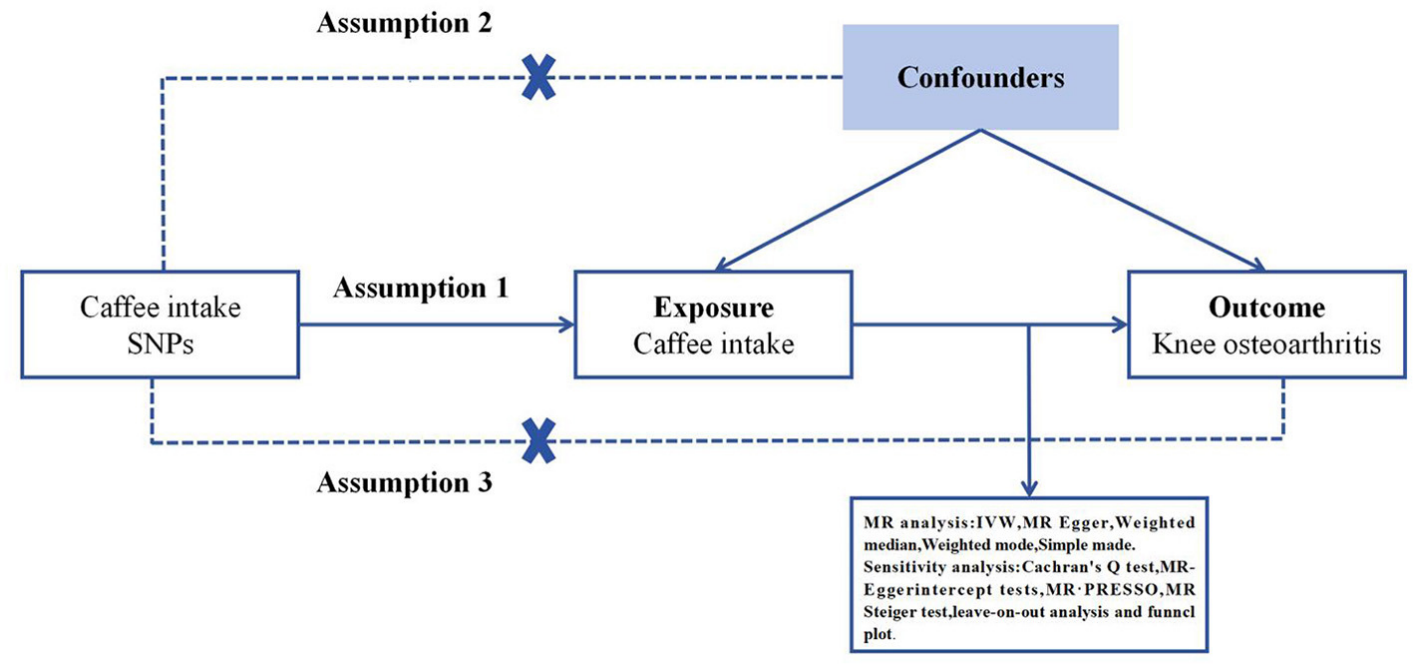
Three key assumptions of the Mendelian randomization study. (1) Genetic variants are significantly associated with exposure coffee intake; (2) the genetic variants remain unaffected by potential confounding factors; (3) the genetic variants can exert their influence on knee osteoarthritis only through coffee intake, without any direct effects. SNP, single nucleotide polymorphism.

Mendelian Randomization (MR) employs genetic variation to estimate the causality between an exposure and an outcome, and its validity is grounded in three hypotheses: (1) the genetic instrumental variables (IVs) are associated with the exposure factors, (2) the IVs are independent of any confounding factors, and (3) the IVs exclusively influence the outcomes through the exposure factors (32) (Figure 1). The fixed-effect inverse variance weighting (IVW), MR-Egger, weighted median, weighted mode, and simple model were employed to assess the causal association, with IVW considered as the primary analytical method (33). The MR-Egger method was employed to evaluate horizontal pleiotropy. An intercept value close to 0 and *p* > 0.05 indicated the absence of horizontal pleiotropy (34). The IVW and MR-Egger methods were employed to quantify the heterogeneity effect between the genetic instruments, and any heterogeneous SNPs were eliminated using the Mendelian Randomization Pleiotropy RESidual Sum and Outlier (MR-PRESSO) packages. F-statistics were utilized to assess the strength of instrumental variables (IVs) in the analysis. The R2 represents the proportion of variance in coffee intake that can be explained by the genetic instruments.

The R2 and F-statistic values for each variable were computed as follows:


$$\begin{array}{l} R^{\text{2}}=2\times \left(1-EAF\right)\times EAF\times {\left(\frac{BETA}{SE\times \sqrt{N}}\right)}^{2} \end{array}$$

$$\begin{array}{l} F=\frac{N-k-1}{k}\times \frac{R^{2}}{1-R^{2}} \end{array}$$

where *k* denotes the number of genetic variants (*k* = 1) and N represents the sample size (35). *F* > 10 indicated that weak IV deviations were unlikely (36).

To further assess the robustness of the results, additional sensitivity analyses were conducted using the leave-one-out plot and funnel plot. All statistical analyses were carried out using the “TwoSampleMR” and “MR-PRESSO” packages in R version 4.3.1. The significance level was set at *p* < 0.05 to determine statistical significance.

## 3 Results

### 3.1 Cross-sectional study

#### 3.1.1 Characteristics of the study participants

A total of 14,580 participants (aged 20 years or older) were included in this analysis and classified into non-OA (*n* = 12,827) and OA (*n* = 1,753) groups, as shown in Table 1. There were significant differences in caffeine intake, age, gender, race, educational level, BMI, activity, drinking status, smoking status, diabetes, hypertension, and depression between the two groups. Compared to the non-OA participants, OA patients were more likely to be female, older, non-Hispanic white, smokers or drinkers, obese, depressed, with low physical activity, a history of hypertension and diabetes, and high caffeine intake.

**Table 1:** Baseline characteristics of study participants with or without osteoarthritis in the NHANES 2009–2016.

**Characteristics**	**Total number of participants (*****n =*** **14,580)**		**P**
	**Non-osteoarthritis**	**Osteoarthritis**	
	***N** = **12,827***	***N** = **1,753***	
**Gender**, ***n*** **(%)**			< 0.001
Female	6,091 (47.5%)	1,106 (63.1%)	
Male	6,736 (52.5%)	647 (36.9%)	
Age (years, mean ± SD)	44.60 ± 16.80	61.56 ± 13.75	< 0.001
**Race/ethnicity**, ***n*** **(%)**			< 0.001
Mexican American	1,993 (15.5%)	158 (9.01%)	
Other Hispanic	1,326 (10.3%)	134 (7.64%)	
Non-Hispanic Black	2,633 (20.5%)	313 (17.9%)	
Non-Hispanic White	5,163 (40.3%)	1,035 (59.0%)	
Other Race - Including Multi-Racial	1,712 (13.3%)	113 (6.45%)	
**Education**, ***n*** **(%)**			0.020
Under high school	2,661 (20.7%)	370 (21.1%)	
High school or equivalent	2,817 (22.0%)	362 (20.7%)	
Some College or AA degree	3,903 (30.4%)	591 (33.7%)	
College Graduate or above	3,446 (26.9%)	430 (24.5%)	
**Marital status**, ***n*** **(%)**			0.114
Married/living with partner	7,728 (60.2%)	1,021 (58.2%)	
Widowed/divorced/ separated/never married	5,099 (39.8%)	732 (41.8%)	
**PIR**, ***n*** **(%)**			0.380
< 1.3	6,522 (50.8%)	862 (49.2%)	
1.3–3.5	3,391 (26.4%)	472 (26.9%)	
>3.5	2,914 (22.7%)	419 (23.9%)	
**Smoked at least 100 cigarettes in life**, ***n*** **(%)**			< 0.001
No	7,568 (59.0%)	803 (45.8%)	
Yes	5,259 (41.0%)	950 (54.2%)	
**Had at least 12 alcohol drinks past 1 year?** ***n*** **(%)**			0.047
No	3,346 (26.1%)	497 (28.4%)	
Yes	9,481 (73.9%)	1,256 (71.6%)	
**Vigorous work activity**, ***n*** **(%)**			< 0.001
No	10,168 (79.3%)	1,496 (85.3%)	
Yes	2,659 (20.7%)	257 (14.7%)	
**PHQ-9 score**, ***n*** **(%)**			< 0.001
0–9	11,936 (93.1%)	1,499 (85.5%)	
≥10	891 (6.95%)	254 (14.5%)	
**Hypertension*****, n*** **(%)**			< 0.001
No	9,292 (72.4%)	683 (39.0%)	
Yes	3,535 (27.6%)	1,070 (61.0%)	
**Body mass index**, ***n*** **(%)**			< 0.001
< 25 kg/m^2^	4,091 (31.9%)	366 (20.9%)	
25–30 kg/m^2^	4,324 (33.7%)	499 (28.5%)	
≥30 kg/m^2^	4,412 (34.4%)	888 (50.7%)	
**Diabetes**, ***n*** **(%)**			< 0.001
No	11,410 (89.0%)	1,262 (72.0%)	
Yes	1,179 (9.19%)	421 (24.0%)	
Borderline	238 (1.86%)	70 (3.99%)	
Caffeine, *n* (%)	142.60 ± 189.19	177.05 ± 226.81	< 0.001
< 11 (mg/day)	3,257 (25.4%)	359 (20.5%)	
11 to < 95 (mg/day)	3,275 (25.5%)	368 (21.0%)	
95 to < 206 (mg/day)	3,183 (24.8%)	479 (27.3%)	
≥206 (mg/day)	3,112 (24.3%)	547 (31.2%)	

NHANES, National Health and Nutrition Examination Survey; SD, standard deviation; Values are mean ± SD or *n* (%).

#### 3.1.2 Association between caffeine intake and risk of osteoarthritis

Table 2 presents the relationship between caffeine intake and OA prevalence in the logistic regression model. The analysis revealed a significant positive association between caffeine intake and the prevalence of OA. For instance, the correlation between high caffeine intake (95–206 and ≥206 mg/day) and OA prevalence remained significantly high even after adjusting for covariates using the lowest caffeine intake (< 11 mg/day) as a reference: Model 1—OR (95%Cl) = 1.365 (1.18–1.58) and 1.59 (1.38–1.83); Model 2—OR (95%Cl) = 1.21 (1.04–1.42) and 1.44 (1.23–1.68); and Model 3—OR (95%Cl) = 1.19 (1.01–1.40) and 1.30 (1.10–1.52), respectively (*p* < 0.05).

**Table 2:** Association between caffeine intake and osteoarthritis (logistic regression model).

**Exposure caffeine intake (mg/day)**	**Model 1 OR (95% CI), P**	**Model 2 OR (95% CI), P**	**Model 3 OR (95% CI), P**
< 11	Reference	Reference	Reference
11 to < 95	1.02 (0.87,1.19) 0.827	1.03 (0.875,1.21) 0.726	1.05 (0.89,1.25) 0.54
95 to < 206	1.365 (1.18,1.58) < 0.001	1.21 (1.04,1.42) 0.015	1.19 (1.01,1.40) 0.03
≥206	1.59 (1.38,1.83) < 0.001	1.44 (1.23,1.68) < 0.001	1.30 (1.10,1.52) 0.002

Non-adjusted model (Model 1): None. Minimally adjusted model (Model 2): adjusted for age, gender, race, educational level; HyperFully adjusted model (Model 3): adjusted for age, gender, race, educational level, BMI, activity, drinking status, smoking status, diabetes, hypertension, and PHQ-9 score.

#### 3.1.3 The dose-response association

**Figure 2:**
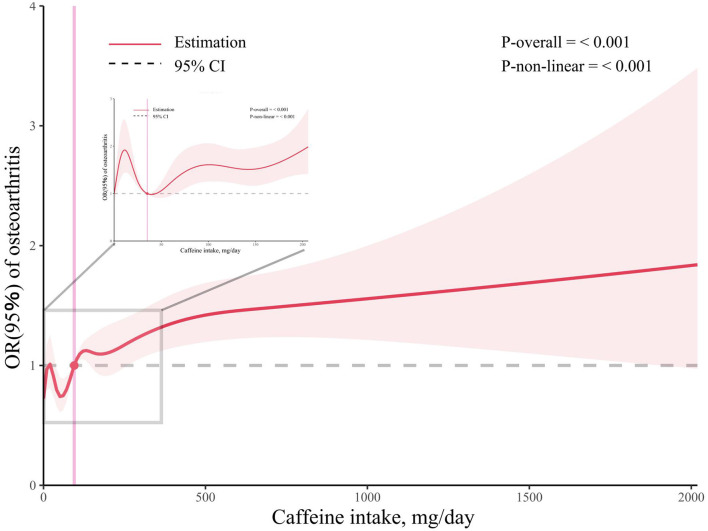
Associations between caffeine intake and the prevalence of osteoarthritis. The enlarged subplot depicts the relationship between 0 milligrams and 206 milligrams.

We employed RCS regression to illustrate the dose-response association between caffeine intake and OA prevalence while adjusting for multiple variables (Figure 2). The results indicated a non-linear (*p* < 0.001) and positive correlation between caffeine intake and OA prevalence, with inflection points at 95 mg/day.

### 3.2 MR study

#### 3.2.1 Selection of genetic IVs for MR

**Figure 3:**
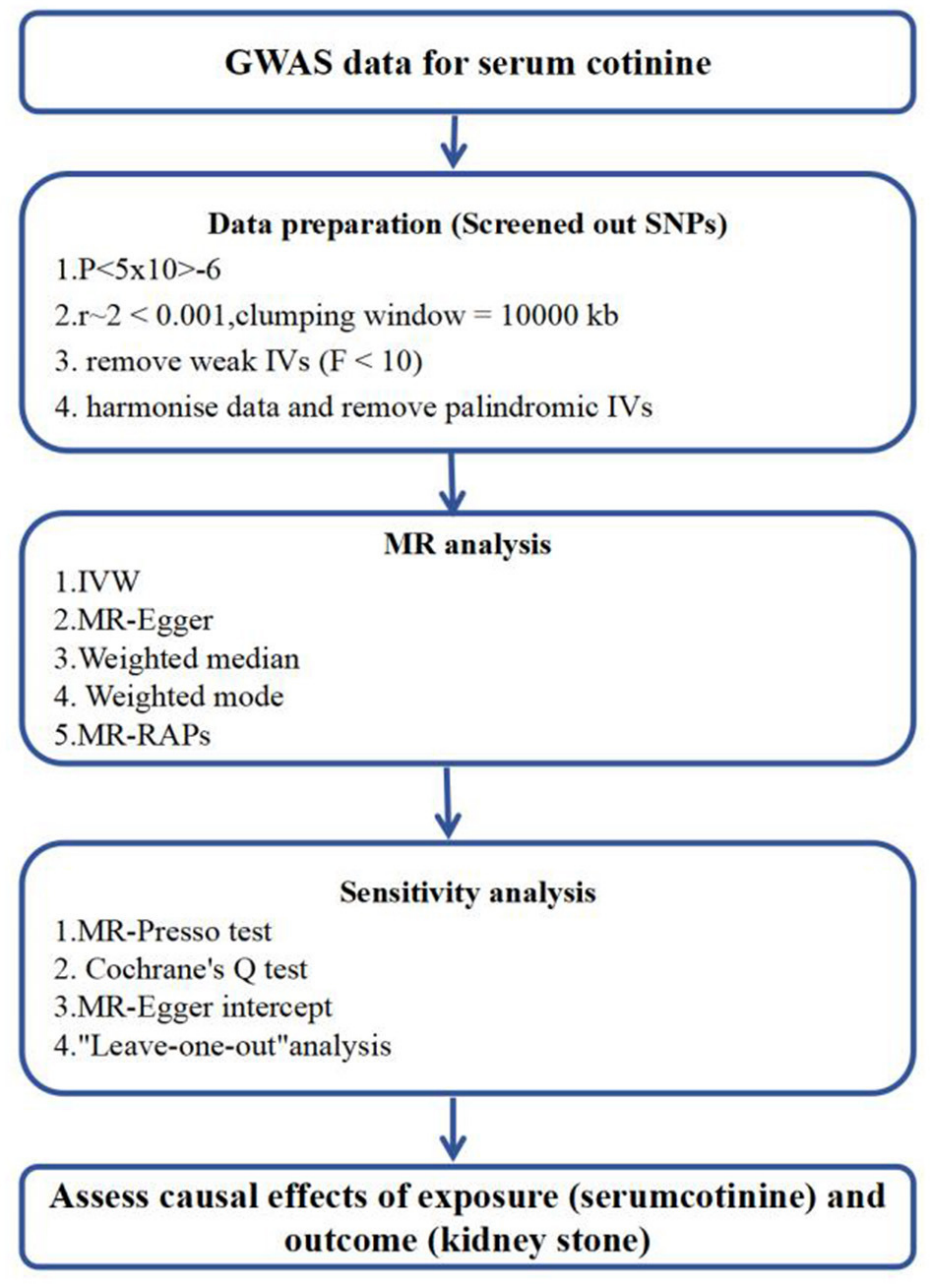
Flowchart of Mendelian Randomization analysis. MR Mendelianrandomization, GWAS genome-wide association study. MR-PRESSO, MR-Pleiotropy RESidual Sum and Outlier; IVW, inverse-variance weighting; MR-RAPS, MR-robust adjusted profilescore.

In the two-sample MR analysis, 22 SNPs associated with coffee intake were identified through linkage disequilibrium analysis (Figure 3). The F statistics of each SNP was >10 (Table 3), and there was a significant correlation with outcome variables, indicating an absence of weak instrument bias. Outliers identified through the MR-PRESSO analysis were excluded, and the SNPs that remained after excluding ambiguous and palindromic SNPs were retained as IVs.

**Table 3:** Characteristics of SNPs associated with coffee intake.

**SNP**	**EA**	**BETA**	**SE**	**P1**	**p2**	**N**	**R2**	**F**
rs1527961	C	−0.0133	0.0024	1.70E-08	0.2519	428860	0.0000742	32
rs12989746	T	0.0104	0.0019	2.80E-08	0.3384	428860	0.0000719	31
rs2597805	T	0.0099	0.0018	2.00E-08	0.5026	428860	0.0000734	31
rs73075167	T	−0.0161	0.0024	5.00E-11	0.6107	428860	0.0001007	43
rs7811609	T	0.0091	0.0017	4.00E-08	0.8114	428860	0.0000703	30
rs4410790	C	0.0391	0.0017	1.20E-120	0.7001	428860	0.0012704	546
rs442355	C	−0.0111	0.0019	1.90E-09	0.9249	428860	0.0000842	36
rs78267637	G	−0.0254	0.0043	3.90E-09	0.5918	428860	0.0000809	35
rs6469262	C	−0.0092	0.0016	1.90E-08	0.3838	428860	0.0000736	32
rs10119174	C	−0.0094	0.0016	1.00E-08	0.9741	428860	0.0000764	33
rs117810762	A	0.0359	0.0062	6.20E-09	0.1158	428860	0.0000788	34
rs61928609	C	−0.0147	0.0022	1.30E-11	0.0000	428860	0.0001069	46
rs117968677	A	−0.0310	0.0055	1.90E-08	0.5986	428860	0.0000738	32
rs8056750	T	0.0105	0.0017	1.30E-09	0.1644	428860	0.0000857	37
rs57918684	A	0.0129	0.0022	8.60E-09	0.0631	428860	0.0000773	33
rs1942965	C	−0.0089	0.0016	3.80E-08	0.9550	428860	0.0000705	30
rs630194	C	−0.0114	0.0017	2.30E-11	0.3476	428860	0.0001042	45
rs56113850	C	0.0127	0.0016	8.90E-15	0.3725	428860	0.0001402	60
rs6063085	C	0.0104	0.0017	4.50E-10	0.4174	428860	0.0000907	39
rs6062682	T	0.0104	0.0016	2.50E-10	0.7838	428860	0.0000933	40
rs13054099	C	−0.0108	0.0018	4.30E-09	0.7693	428860	0.0000803	34
rs17842490	G	−0.0452	0.0068	3.30E-11	0.9151	428860	0.0001026	44

SNP, singlE–nucleotide polymorphism; EAF, effect allele frequency; EA, effect allele; BETA, beta.exposure; SE, standard error; P1, pval. coffee; P2, pval. Knee osteoarthritis; R^2^ was calculated as follows: $R^{\text{2}}=2\times \left(1-EAF\right)\times EAF\times {\left(\frac{BETA}{SE\times \sqrt{N}}\right)}^{2}$. The F-statistic for each SNP was calculated as follows: $F=\frac{N-k-1}{k}\times \frac{R^{2}}{1-R^{2}}$. 22 SNPs were included for MR analyses.

#### 3.2.2 Causal effects of caffeine intake on osteoarthritis

**Figure 4:**
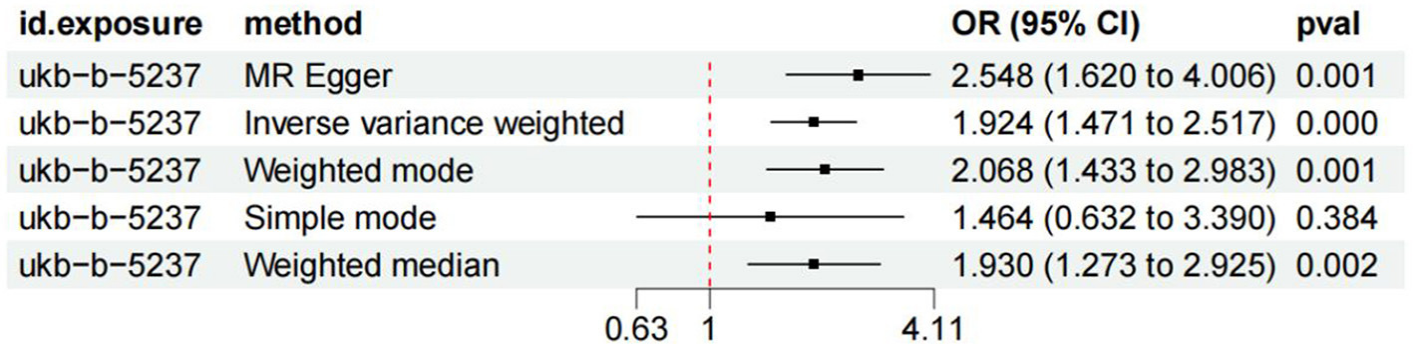
Forest plots of MR study using genetically predicted coffee intake with knee osteoarthritis. IVW, MR-Egger, weighted median, weighted mode, and simple mode were used in this study.

**Figure 5:**
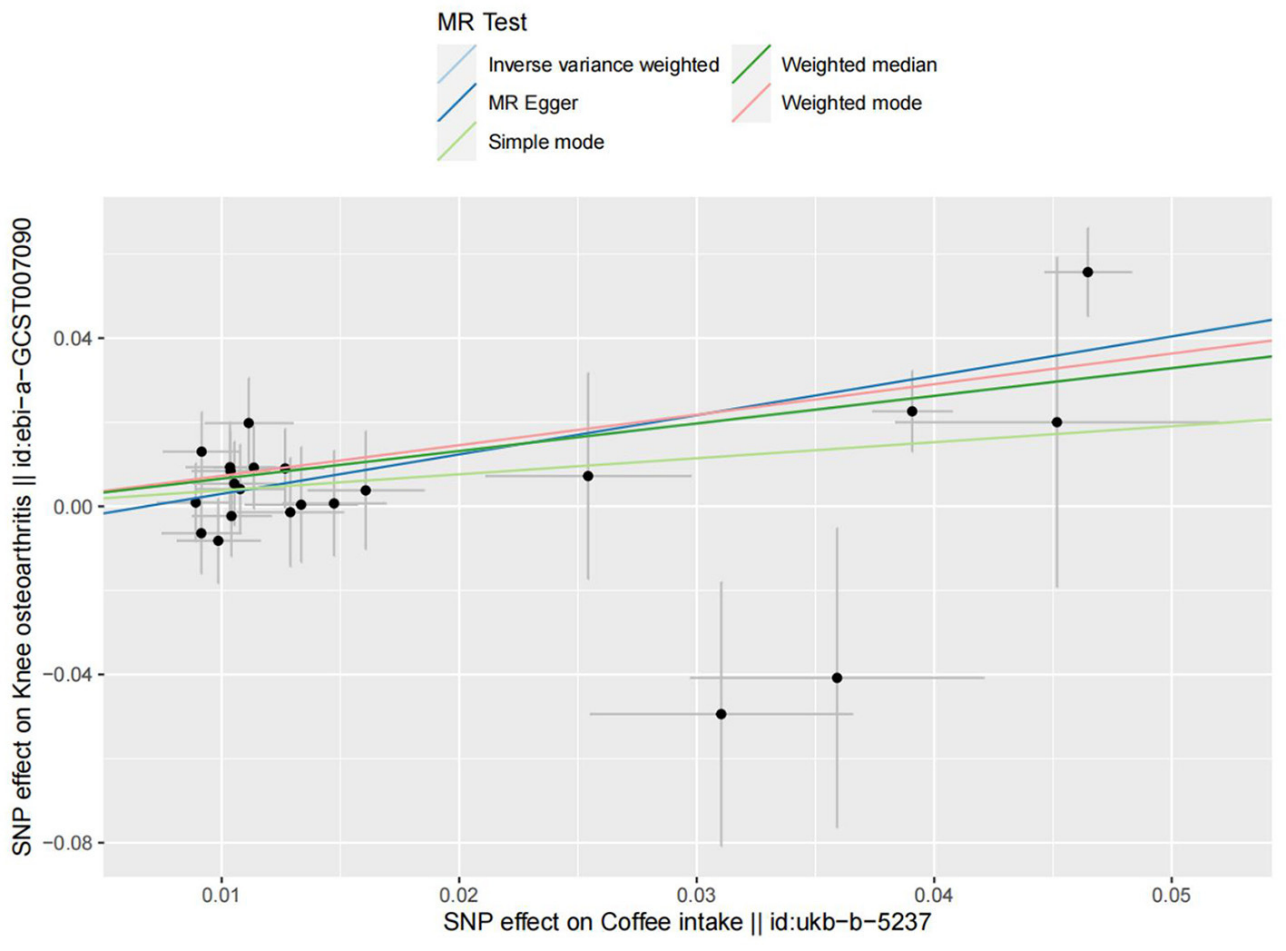
The scatter plot for MR analyses of causal associations between each coffee intake SNP and knee osteoarthritis.

The fixed-effect IVW analysis results indicated a significant association between coffee consumption and the risk of knee osteoarthritis [OR (95% CI) = 1.94 (1.471–2.517), *p* < 0.001] and consistent findings were also observed in the MR-Egger regression, weighted median, weighted mode, and simple mode analyses, supporting the significant effect of coffee intake on the risk of knee osteoarthritis (Figures 4, 5). The leave-one-out sensitivity analysis demonstrated that the overall effects remained unchanged or reversed when any single SNP was removed, indicating the credibility of the results (Supplementary Figure S2). The forest plots displaying the estimates of the association between coffee intake and knee osteoarthritis for each SNP can be found in Supplementary Figure S3.

#### 3.2.3 Sensitivity analysis

The heterogeneity detected in some outcomes does not invalidate MR results (Table 4). This is due to the employment of the random-effects IVW used in this study, which effectively mitigates pooled heterogeneity. Additionally, our analysis of funnel plots revealed that the effect-size variations around the point estimates were generally symmetric, indicating an absence of horizontal pleiotropy in the analysis (Supplementary Figure S4). In the MR-PRESSO global test and MR-Egger intercept test, all *p*-values exceeded 0.05, indicating the absence of horizontal pleiotropy in the analysis.

**Table 4:** Sensitivity analysis of the causal relationship between coffee consumption and the risk of knee osteoarthritis.

**Outcomes**	**Heterogeneity test**						**Pleiotropy test**		
	**MR-Egger**			**Inverse variance weighted**			**MR-Egger**		
	**Q**	**Q_df**	**Q_** ***pval***	**Q**	**Q_df**	**Q_** ***pval***	**Intercept**	**SE**	***p***
Spontaneous abortion	14.4	19	0.761	14.4	20	0.811	0	0.005	0.999

df, degree of freedom; MR, Mendelian randomization; Q, heterogeneity statistic Q.

## 4 Discussion

The present study showed a positive association between OA and daily caffeine intake of over 95 mg. However, the results varied with age, gender, race, educational level, BMI, physical activity, drinking status, smoking status, diabetes, hypertension, and PHQ-9 score.

A previous study suggested that caffeine may become harmful at a particular concentration (37). Excessive and persistent caffeine consumption in adults (500–600 mg/day, equivalent to 4–7 cups of coffee) has been associated with a range of health problems. These may include feelings of nervousness and irritability, difficulty sleeping, irregular heartbeat, increased urine production, rapid breathing, digestive issues, elevated calcium levels in urine, reduced fertility in women, as well as an elevated risk of osteoporosis and hip fractures (21, 38). Furthermore, various studies conducted both in laboratory settings and on living organisms have indicated that excessive caffeine consumption can have detrimental effects on the musculoskeletal system, specifically on tissues such as the hyaline cartilage (23–25, 27).

**Figure 6:**
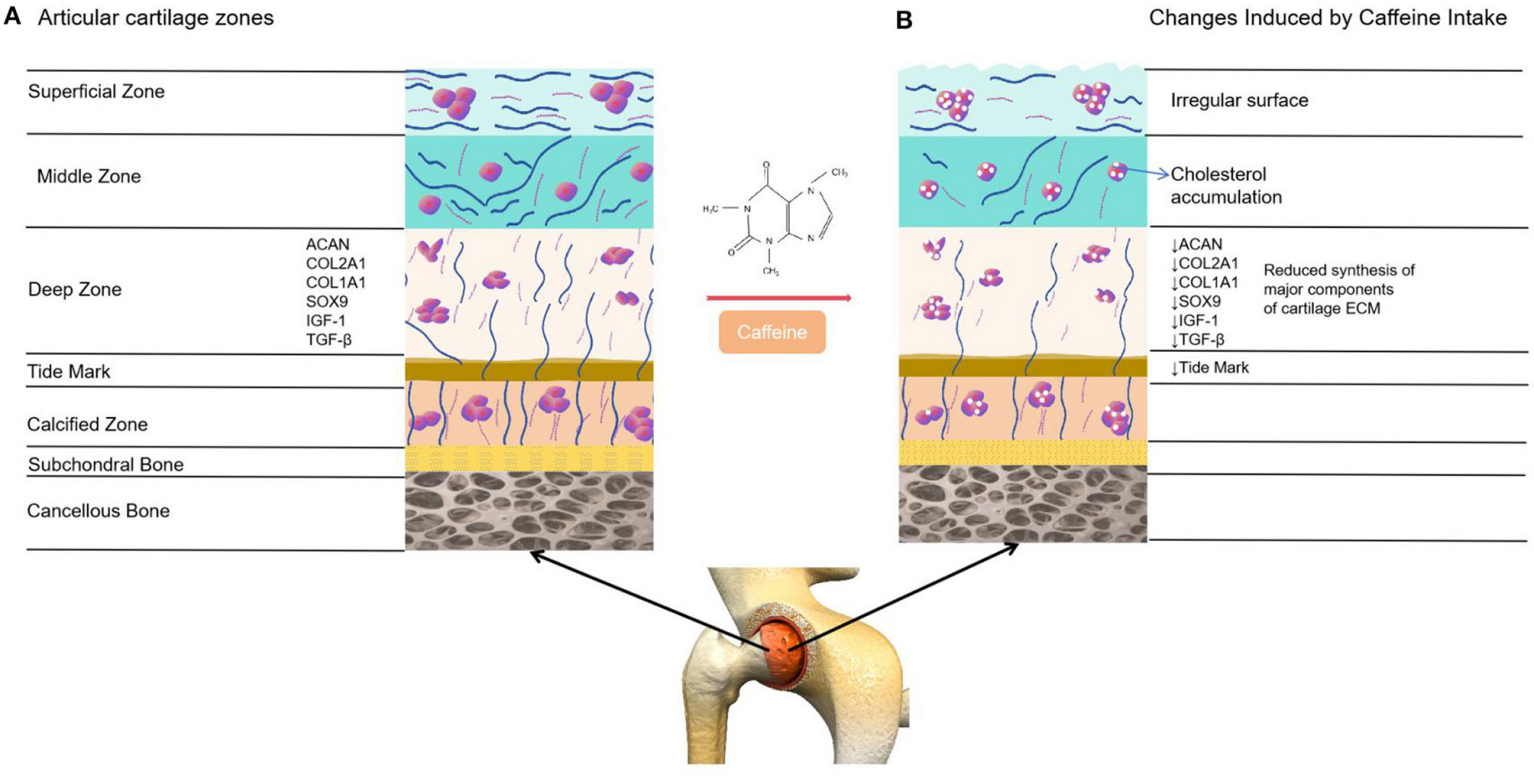
Comparison between healthy articular cartilage and the changes induced on it by caffeine. **(A)** Normal articular cartilage appearance. Articular cartilage is composed of chondrocytes and its ECM. They respond to a variety of stimuli, such as cytokines, mechanical loading and growth factors. Among these, insulin growth factor 1 (IGF-1) and Transforming Growth Factor Beta 1 (TGF-ß1) are involved in cartilage homeostasis and chondrocyte responses to mechanical loading. Likewise, a wide and remarkably tidemark is observed, as well as a regular surface that provides the ideal biomechanical properties to the joint. **(B)** The articular cartilage changes induced by caffeine intake. This alkaloid reduces the synthesis of major cartilage ECM components. It also diminishes chondrocyte proliferation, decreases the tidemark and is associated with an irregular surface of the superficial zone of the cartilage. Additionally, caffeine is linked to lower chondrocyte quality due to cholesterol accumulation.

Articular cartilage, a form of hyaline cartilage, serves as a protective covering on the articulating surfaces of synovial joints (5, 6). Due to the absence of nerves, blood vessels, and lymphatic vessels, this tissue has limited capacity for self-repair (39). Articular cartilage is made up of inactive chondrocytes and an extracellular matrix (ECM) consisting mainly of water, collagens (including collagen type I alpha 1 and collagen type II alpha 1, known as COL2A1), and proteoglycans (such as aggrecan, also referred to as ACAN) (39). The precise makeup of the cartilage extracellular matrix is vital for preserving its distinctive mechanical characteristics and is crucial for the survival of chondrocytes (5, 6). An experimental study conducted on live rats revealed the potential impact of caffeine consumption on the articular cartilage (20, 26). A separate investigation discovered that consuming caffeine at concentrations ranging from 1 to 100 μM led to a decrease in the mRNA expression of critical extracellular matrix components (such as COL2A1 and ACAN) in articular cartilage cells (40). Furthermore, the consumption of caffeine also diminishes the mRNA expression of various members involved in the IGF-1 signaling pathway, such as IGF1, IGF1-receptor, and AKT, which play a crucial role in promoting anabolic responses in chondrocytes (41). Moreover, the consumption of caffeine leads to a reduction in chondrocyte proliferation and is linked with a decrease in the tidemark as well as the presence of surface irregularities in the superficial zone of the cartilage (40) (Figure 6).

In this study, it was observed that in comparison to non-OA patients, OA patients exhibited a higher likelihood of being female, older, non-Hispanic white, smokers or drinkers, having low physical activity, being obese, experiencing depression, with a history of hypertension and diabetes, and having high caffeine intake. Recently, Mendelian randomization (MR) has gained popularity as a highly effective method for analyzing causal inference. It leverages genetic variation as an instrumental variable (IV) to determine causality between the outcome and exposure, providing an effective means to mitigate the confounding bias often present in traditional epidemiological studies (42). In this research, we addressed bias concerns by selecting a GWAS dataset featuring significant coffee intake and knee OA samples, from which we screened 23 SNPs. The causality between these two sample sets was analyzed using the IVW, MR-Egger, weighted median, weighted mode, and simple mode methods to ensure comprehensive assessment. The findings demonstrated a consistent association between coffee consumption and an increased susceptibility to knee osteoarthritis, suggesting a causal relationship.

One notable strength of this study is its robustness, as it leverages a large cross-sectional dataset from NHANES and employs a two-sample Mendelian randomization (MR) analysis. Cross-sectional studies provide a means to investigate the association between caffeine intake and OA risk at the population level through self-reported data. Mendelian randomization (MR) addresses the limitations of traditional epidemiological studies, such as residual confounding, reverse causation, and measurement errors, thereby offering a more robust analytical approach. Nevertheless, the current study has certain constraints. As a cross-sectional study using a database, it was only possible to evaluate the correlation between caffeine intake and OA, without accurately demonstrating a causal relationship. Furthermore, this study had limitations in terms of the number of confounding factors, which was limited to 13, and the sample size, with only 14,580 individuals being assessed, since only four cycles of the NHANES survey (2009–2016) were analyzed and several samples with incomplete data were excluded. Moreover, it is important to note that this study did not involve clinical evaluations of OA, relying instead on NHANES interview data to determine the occurrence of OA in patients, which may introduce inaccuracies. Additionally, the study did not account for medication use in relation to caffeine intake, despite the inclusion of certain medications that contain caffeine (42). The NHANES data did not provide information on the specific amount of caffeine in the drinking water across different regions. Moreover, the majority of participants in the NHANES were non-Hispanic white, which limits the generalizability of the study's findings to other populations. Lastly, this study relied on self-reported questionnaires rather than utilizing objective biological measures to estimate coffee intake. Research has revealed a weak correlation between self-reported questionnaires and actual caffeine intake, highlighting the discrepancy between questionnaire-based data and biological measurements. Accurate details regarding coffee consumption, such as brewing method, coffee type, and quantity, are crucial for investigating the potential relationship between caffeine intake and exposure to genes. Consequently, it is essential to take these factors into account in future analyses of caffeine intake and the prevalence of OA.

## 5 Conclusion

The findings of our study indicated a direct link between the prevalence of OA and high levels of caffeine intake (>95 mg/day). In order to mitigate the adverse impacts of caffeine, it is essential to regulate and limit its consumption. It is particularly crucial to closely monitor and restrict caffeine intake for specific individuals, such as infants and pregnant women, who have decreased metabolism for this substance. Consequently, healthcare professionals, including doctors and nurses, should possess a comprehensive understanding of the potential risks caffeine poses to the musculoskeletal system and offer appropriate guidance to their patients. Table 5 provides a comprehensive list of food and beverage items that have a caffeine content of ≥95 mg.

**Table 5:** The consumption of food that contains 95 mg of caffeine (data provided by the USDA's food and nutrient database for dietary studies).

**Food and beverages**	**Consumption**
Not reconstituted instant coffee	3.03 g
Reconstituted instant coffee	365.38 g
Espresso	44.80 g
Brewed coffee	237.50 g
Mocha coffee	287.88 g
Cappuccino	263.88 g
Green tea	791.66 g
Black tea	475.00 g
Black chocolate	169.64 g
Regular cola	1,055.55 g
Diet cola	791.66 g
Energy drink (Red Bull)	327.58 g

## Data availability statement

Publicly available datasets were analyzed in this study. This data can be found here: www.cdc.gov/nchs/nhanes/.

## Ethics statement

Ethical review and approval was not required for the study on human participants in accordance with the local legislation and institutional requirements. The patients/participants provided their written informed consent to participate in this study. The studies were conducted in accordance with the local legislation and institutional requirements. Written informed consent for participation was not required from the participants or the participants' legal guardians/next of kin in accordance with the national legislation and institutional requirements.

## Author contributions

ZJ: Writing – original draft, Writing – review & editing, Data curation, Formal analysis, Methodology, Software, Visualization. YS: Data curation, Formal analysis, Writing – original draft, Writing – review & editing. DC: Data curation, Writing – original draft, Writing – review & editing, Investigation. ZL: Data curation, Writing – original draft, Writing – review & editing, Methodology. JD: Formal analysis, Writing – review & editing. BD: Data curation, Writing – original draft, Writing – review & editing, Conceptualization, Formal analysis. WD: Conceptualization, Writing – original draft, Writing – review & editing.

## Supplementary material

Supplementary Figure 1 Flow chart of sample selection from the NHANES 2009–2016.

Supplementary Figure 2 The funnel plot for MR analyses of causal associations between each coffee intake SNP and knee osteoarthritis.

Supplementary Figure 3 Leave-one-out sensitivity analysis for knee osteoarthritis using SNP associated coffee intake.

Supplementary Figure 4 The forest plot for MR analyses of causal associations between each coffee intake SNP and knee osteoarthritis.
