# Supplementary figures and images for: Effect of caffeine intake on self-reported and genetic prediction of osteoarthritis: an epidemiological study and Mendelian randomization analysis
Source: Front Nutr. 2024 Jul 17;11:1405161. doi: 10.3389/fnut.2024.1405161 (PMC11288902; doi:10.3389/fnut.2024.1405161)

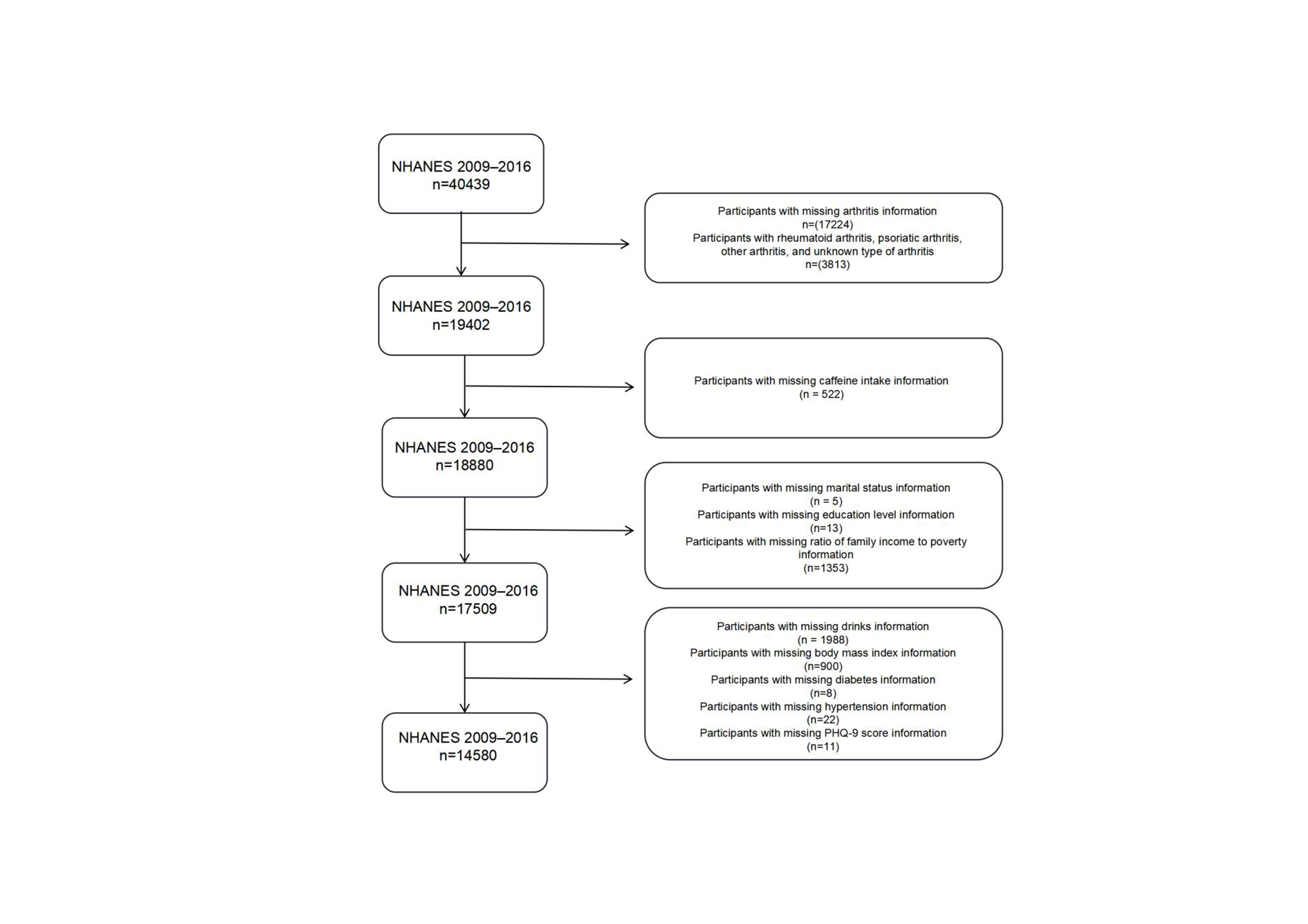

Supplement: Supplementary Figure 1 — Flow chart of sample selection from the NHANES 2009–2016. [file Image_1.JPEG]

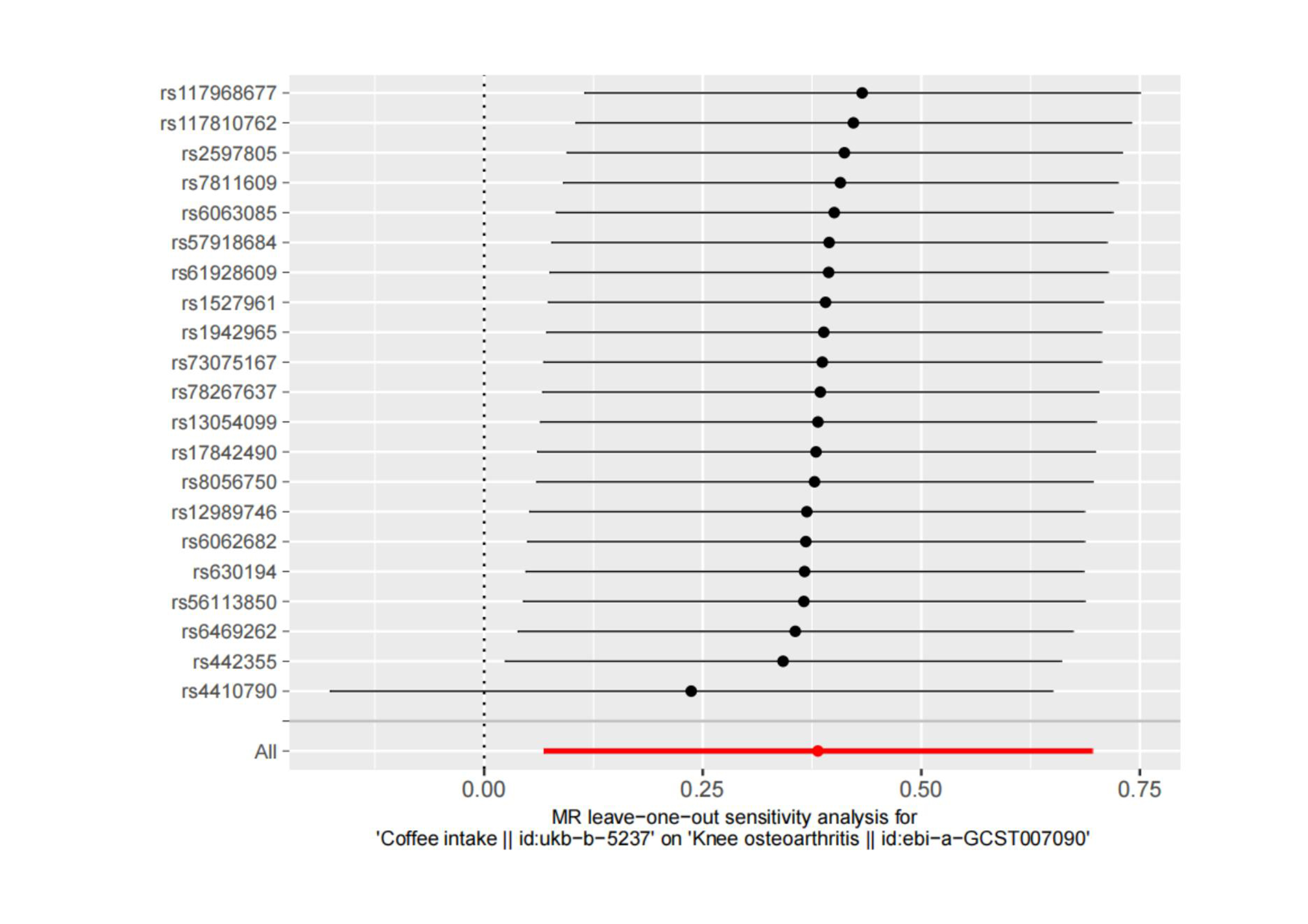

Supplement: Supplementary Figure 2 — The funnel plot for MR analyses of causal associations between each coffee intake SNP and knee osteoarthritis. [file Image_2.JPEG]

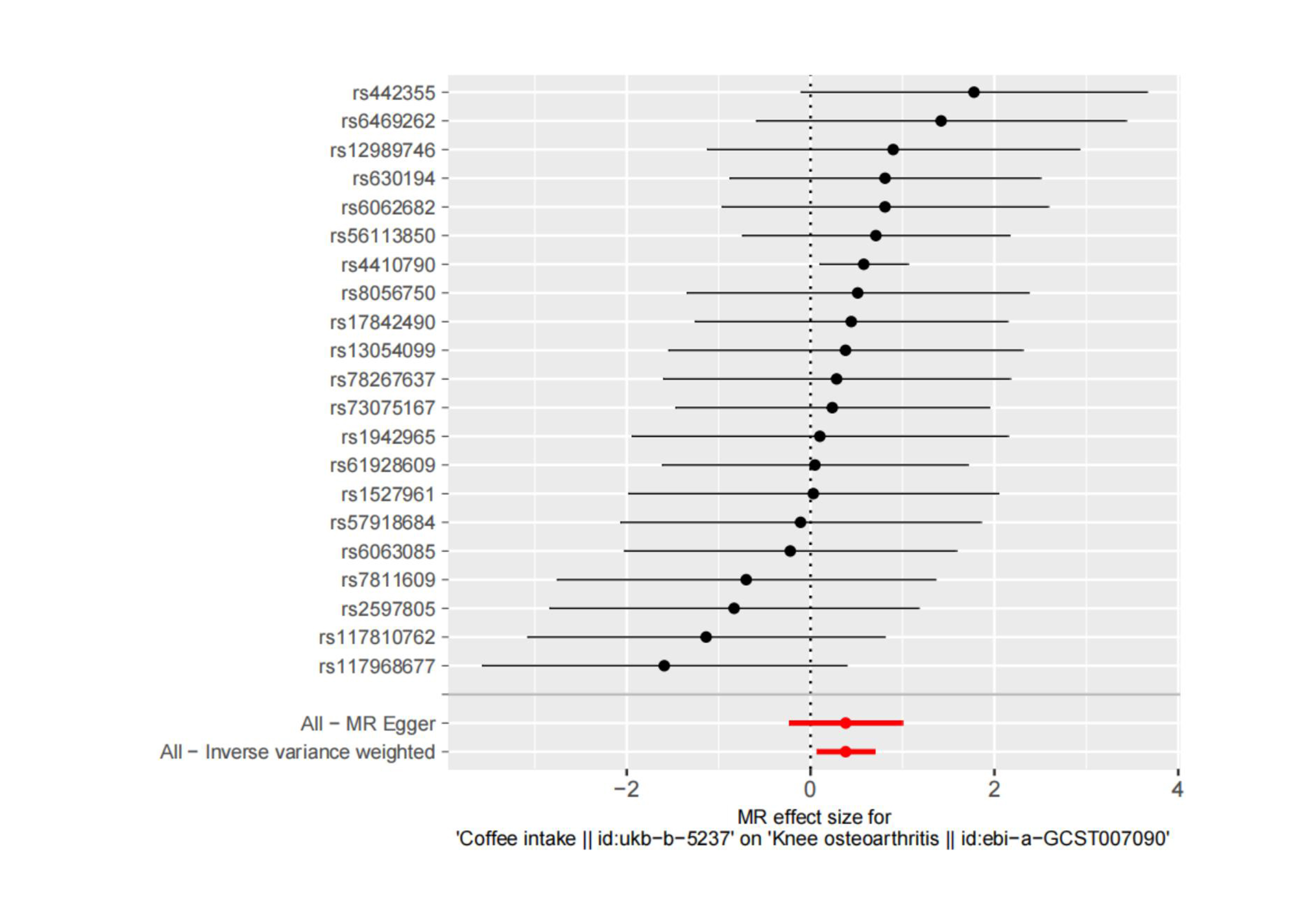

Supplement: Supplementary Figure 3 — Leave-one-out sensitivity analysis for knee osteoarthritis using SNP associated coffee intake. [file Image_3.JPEG]

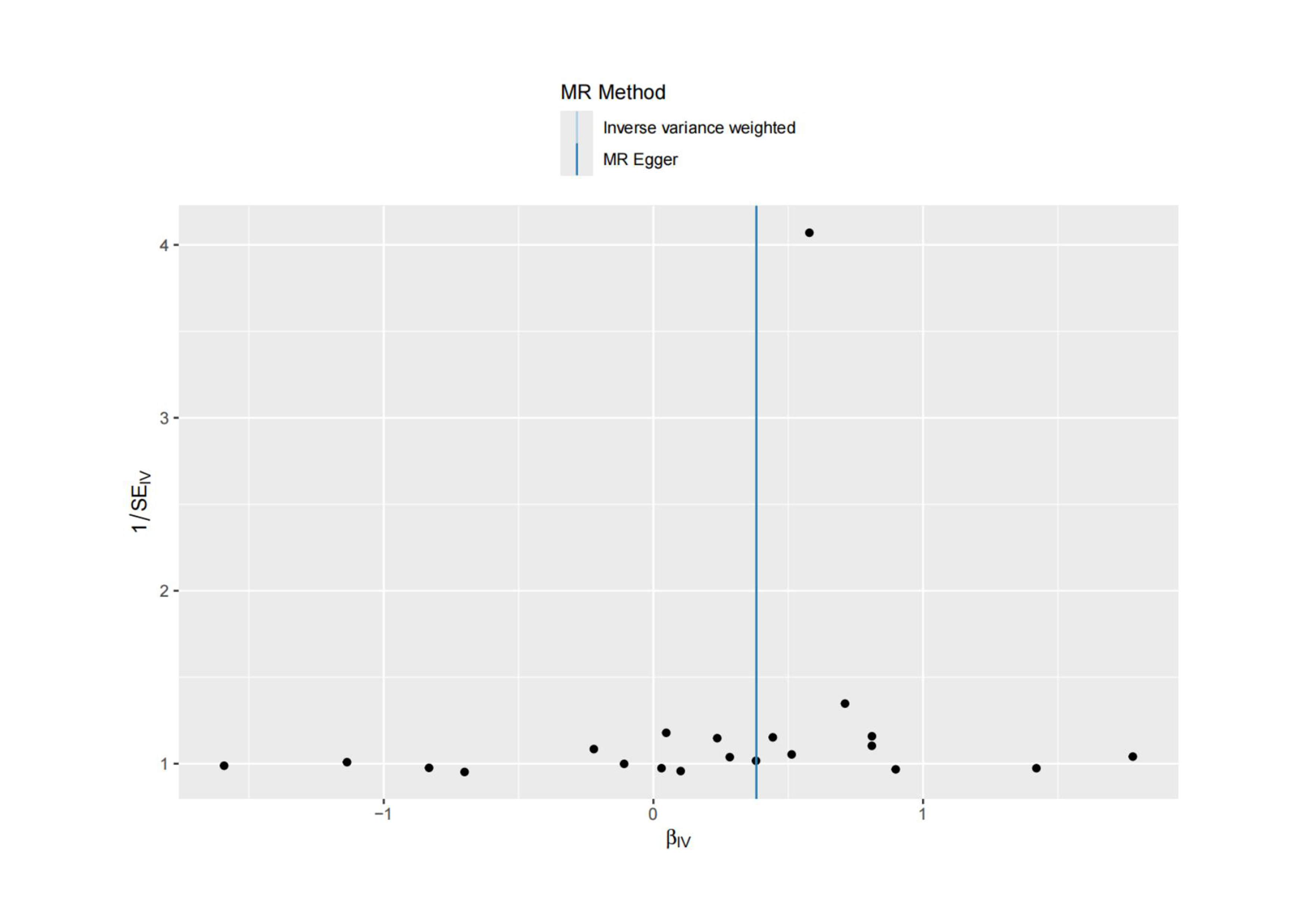

Supplement: Supplementary Figure 4 — The forest plot for MR analyses of causal associations between each coffee intake SNP and knee osteoarthritis. [file Image_4.JPEG]
